# Supplementary material for: Fast non-Abelian geometric gates via transitionless quantum driving
Source: Sci Rep. 2015 Dec 21;5:18414. doi: 10.1038/srep18414 (PMC4685308; doi:10.1038/srep18414)
Supplement: Supplementary Information [file srep18414-s1.pdf]

Supplementary Methods for
"Fast non-Abelian geometric gates via transitionless quantum driving"

J. Zhang,[1,2] Thi Ha Kyaw,[2] D. M. Tong,[1,*] Erik Sjöqvist,[3,4,†] and Leong-Chuan Kwek[2,5,6,7,‡]

[1]*Department of Physics, Shandong University, Jinan 250100, China*
[2]*Centre for Quantum Technologies, National University of Singapore, 3 Science Drive 2, Singapore 117543, Singapore*
[3]*Department of Quantum Chemistry, Uppsala University, Box 518, Se-751 20 Uppsala, Sweden*
[4]*Department of Physics and Astronomy, Uppsala University, Box 516, Se-751 20 Uppsala, Sweden*
[5]*MajuLab, CNRS-UNS-NUS-NTU International Joint Research Unit, UMI 3654, Singapore*
[6]*Institute of Advanced Studies, Nanyang Technological University, 60 Nanyang View, Singapore 639673, Singapore*
[7]*National Institute of Education, Nanyang Technological University, 1 Nanyang Walk, Singapore 637616, Singapore*
(Dated: November 12, 2015)

DEGENERATE TRANSITIONLESS QUANTUM DRIVING ALGORITHM

We consider a degenerate subspace based on a set of bases $\{|\varphi_l^n\rangle, l = 1, ..., m\}$ for an energy E_n. The system Hamiltonian H_0 can be written as

$$H_0(t) = \sum_n E_n \sum_{l=1}^{m} |\varphi_l^n\rangle\langle\varphi_l^n|. \tag{S1}$$

When the adiabatic condition is satisfied, there will be no level cross between subspaces with different energies. Therefore, we consider the n-th subspace with eigen-energy E_n for simplicity. The states that evolve according to Schrödinger equation are labeled as $|\tilde{\varphi}_l^n\rangle$ with initial condition $|\tilde{\varphi}_l^n(0)\rangle = |\varphi_l^n(0)\rangle$. We assume that $|\tilde{\varphi}_l^n\rangle$ and $|\varphi_l^n\rangle$ are related by a basis transformation

$$|\tilde{\varphi}_l^n\rangle = \sum_j |\varphi_j^n\rangle C_{jl}^n \tag{S2}$$

from which we obtain $\langle\varphi_j^n|\frac{\partial}{\partial_t}|\tilde{\varphi}_k^n\rangle = -i\langle\varphi_j^n|H|\tilde{\varphi}_k^n\rangle = -iE_n C_{jk}^n$.

On the other hand, we can show this in another way,

$$\begin{aligned}\langle\varphi_j^n|\frac{\partial}{\partial_t}|\tilde{\varphi}_k^n\rangle &= \langle\varphi_j^n|\frac{\partial}{\partial_t}(\sum_l |\varphi_l^n\rangle C_{lk}^n) \\ &= \sum_l \langle\varphi_j^n|\dot{\varphi}_l^n\rangle C_{lk}^n + \dot{C}_{jk}^n\end{aligned} \tag{S3}$$

which implies that

$$\dot{C}_{jk}^n = -iE_n C_{jk}^n - \sum_l A_{jl}^n C_{lk}^n \tag{S4}$$

where $A_{jl}^n = \langle\varphi_j^n|\dot{\varphi}_l^n\rangle$. Now, we obtain the transition relationship between the two sets of basis, which is

$$C^n = T\exp(-\int A^n \mathrm{d}t)\exp(-i\int E_n \mathrm{d}t) \tag{S5}$$

It is evident to see that the second part of C^n is the dynamical phase that is the same as in non-degenerate case, while the first part is a degenerate analog of the Abelian geometric phase.

By using the expression of C^n, we can write the time evolution operator of this adiabatically evolved system as

$$U^n(t) = \sum_l |\tilde{\varphi}_l^n\rangle\langle\varphi_l^n(0)| \tag{S6}$$

According to Schrödinger equation, the required Hamiltonian to achieve this time evolution reads

$$\begin{aligned} H^n(t) &= i\dot{U}^n(t)U^{n-1}(t) \\ &= i\sum_l (\frac{d}{dt}|\tilde{\varphi}_l^n\rangle)\langle\tilde{\varphi}_l^n| \\ &= \sum_l (E_n|\varphi_l^n\rangle\langle\varphi_l^n| + i|\partial_t\varphi_l^n\rangle\langle\varphi_l^n|) \\ &\quad -i\sum_{l,k} A_{lk}^n|\varphi_l^n\rangle\langle\varphi_k^n| \end{aligned} \tag{S7}$$

Therefore, the total Hamiltonian to drive the system is $H(t) = \sum_n H^n(t)$.

NON-DEGENERATE SHORTCUT

The eigenvalues and eigenstates of H_0^P read

$$\begin{aligned} E_+ &= -E_- = \Omega, E_0 = 0 \\ |E_+\rangle &= \frac{1}{\sqrt{2}}(|e\rangle - \sin\frac{\theta}{2}e^{-i\varphi}|1\rangle + \cos\frac{\theta}{2}|a\rangle) \\ |E_-\rangle &= \frac{1}{\sqrt{2}}(-|e\rangle - \sin\frac{\theta}{2}e^{-i\varphi}|1\rangle + \cos\frac{\theta}{2}|a\rangle) \\ |D\rangle &= |E_0\rangle = \cos\frac{\theta}{2}|1\rangle + \sin\frac{\theta}{2}e^{i\varphi}|a\rangle \end{aligned} \tag{S8}$$

The additional Hamiltonian H_1^P reads

$$\begin{aligned} &i\sum_n |\partial_t n\rangle\langle n| = \\ &\sin^2(\frac{\theta}{2})\dot{\varphi}|1\rangle\langle 1| - (\frac{\dot{\varphi}}{2}\sin\theta e^{-i\varphi} + \frac{i}{2}\dot{\theta}e^{-i\varphi})|1\rangle\langle a| \\ &(-\frac{\dot{\varphi}}{2}\sin\theta e^{i\varphi} + \frac{i}{2}\dot{\theta}e^{i\varphi})|a\rangle\langle 1| - \sin^2(\frac{\theta}{2})\dot{\varphi}|a\rangle\langle a| \end{aligned} \tag{S9}$$

$$-i\sum_n A_n|n\rangle\langle n| =$$

$$\frac{1}{2}\dot{\varphi}\sin^2\frac{\theta}{2}(-|e\rangle\langle e| + (-1+3\cos^2\frac{\theta}{2})|1\rangle\langle 1| + \tag{S10}$$

$$\frac{3}{4}\sin\theta(e^{-i\varphi}|1\rangle\langle a| + H.c) + (3\sin^2\frac{\theta}{2} - 1)|a\rangle\langle a|). \tag{S11}$$

DEGENERATE SHORTCUT

The eigenvalues and eigenstates of H_0^B read

$$\begin{aligned} E_+ &= -E_- = \Omega, E_0^1 = E_0^2 = 0 \\ |E_+\rangle &= \frac{1}{\sqrt{2}}(|e\rangle + \sin\theta\cos\varphi|0\rangle + \sin\theta\sin\varphi|1\rangle + \cos\theta|a\rangle) \\ |E_-\rangle &= \frac{1}{\sqrt{2}}(-|e\rangle + \sin\theta\cos\varphi|0\rangle + \sin\theta\sin\varphi|1\rangle + \cos\theta|a\rangle) \\ |D_1\rangle &= |E_0^1\rangle = \cos\theta(\cos\varphi|0\rangle + \sin\varphi|1\rangle) - \sin\theta|a\rangle \\ |D_2\rangle &= |E_0^2\rangle = \cos\varphi|1\rangle - \sin\varphi|0\rangle \end{aligned} \tag{S12}$$

The shortcut Hamiltonian H_1^B reads

$$
\begin{aligned}
& i\sum_n |\partial_t E_n\rangle\langle E_n| = \\
& i\dot{\varphi}(-|0\rangle\langle 1| + |1\rangle\langle 0|) + i\dot{\theta}\cos\varphi(|0\rangle\langle a| - |a\rangle\langle 0|) \\
& +i\dot{\theta}\sin\varphi(|1\rangle\langle a| - |a\rangle\langle 1|).
\end{aligned} \tag{S13}
$$

$$
\begin{aligned}
& -i\sum A_{ij}^n|\varphi_i^n\rangle\langle\varphi_j^n| = \\
& i\dot{\varphi}\cos\theta\begin{pmatrix} 0 & \cos\theta & -\sin\theta\sin\varphi \\ -\cos\theta & 0 & \sin\theta\cos\varphi \\ \sin\theta\sin\varphi & -\sin\theta\cos\varphi & 0 \end{pmatrix}
\end{aligned} \tag{S14}
$$

* Electronic address: tdm@sdu.edu.cn
† Electronic address: erik.sjoqvist@physics.uu.se
‡ Electronic address: cqtklc@nus.edu.sg
